# Actinomyces Infection Mimicking Esophageal Cancer

**DOI:** 10.7759/cureus.17266

**Published:** 2021-08-17

**Authors:** Jessica J Biller, Ruth Cho, Stanley Zagorski

**Affiliations:** 1 Surgery, Conemaugh Memorial Medical Center, Johnstown, USA

**Keywords:** endoscopy, actinomyces infections, esophageal infection, gastroenterology and endoscopy, infection mimicking malignancy

## Abstract

Actinomycosis is a bacterial infection, which rarely affects the esophagus. Our patient presented with persistent acute blood loss anemia and epigastric pain despite previously negative upper endoscopy. He underwent repeat endoscopy a few months later showing what was thought to be malignant esophageal cancer at the gastroesophageal junction; however, the biopsy report revealed chronic inflammation with actinomycosis. This report will discuss the evaluation and management of actinomyces infections as it is important to distinguish infection from malignancy. It is crucial for physicians to be aware of the unusual presentation and ability to mimic malignancy to aid in proper diagnosis and management and therefore the prevention of unnecessary procedures including resection.

## Introduction

This article was previously presented at Southeastern Surgical Congress in February 2020 as a poster presentation. Actinomycosis is an uncommon infection caused by anaerobic, filamentous bacteria. These infections are most commonly found in the gastrointestinal or genitourinary tracts of immunocompromised patients and are often mistaken for other types of infection or malignancy such as tuberculosis or adenocarcinoma. With proper diagnosis, the treatment consists of prolonged antibiotics; however, abscess drainage or debridement of necrotic tissue may be necessary for some patients [1]. We present a case of an immunocompetent patient who presented with anemia and was found to have esophageal actinomycosis.

## Case presentation

**Figure 1:**
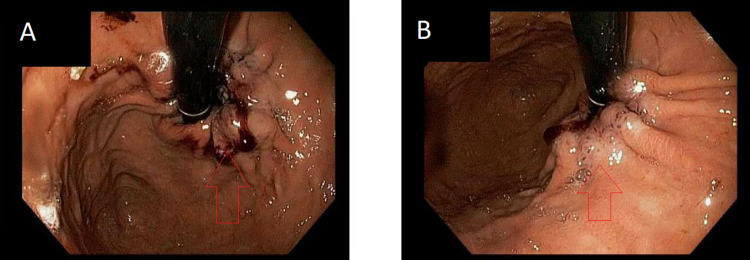
Upper endoscopy showing ulcerated and necrotic tissue at the gastroesophageal junction (A and B near red arrows) in our patient

**Figure 2:**
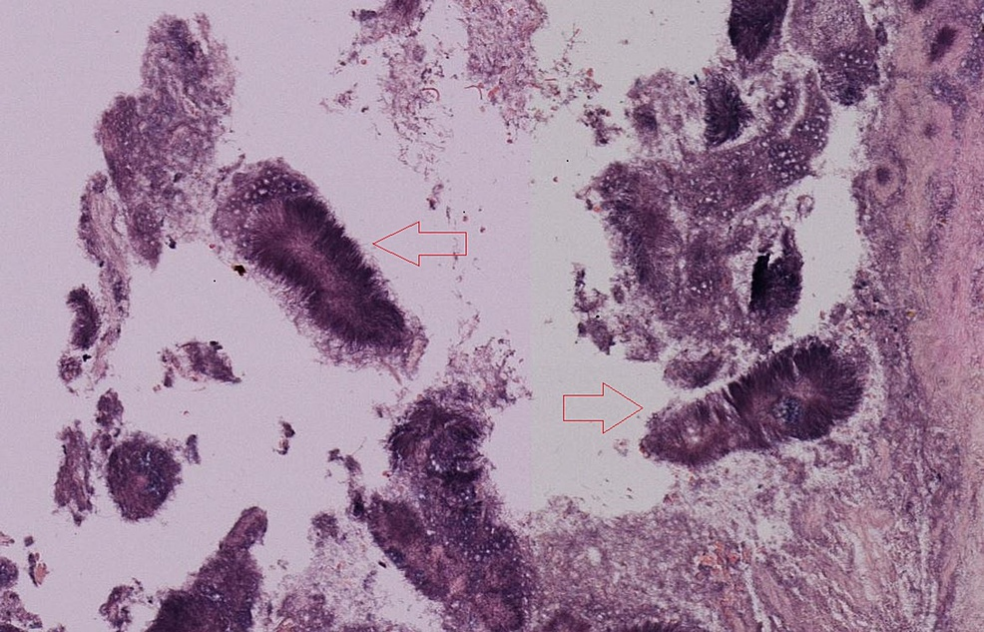
Gram stain showing fragments of columnar epithelial mucosa with chronic active inflammation, actinomycetes colonies (red arrows) in the background of necrotic material

An 84-year-old male with multiple co-morbidities including history of colon cancer, currently on anticoagulation for an aortic valve replacement and atrial fibrillation, initially presented with acute blood loss anemia and complaints of epigastric pain. He underwent esophagogastroduodenoscopy and colonoscopy which were unremarkable except for a small cecal polyp and gastritis. He continued to have blood loss anemia and complaints of epigastric pain so endoscopy was repeated a few months later. On repeat endoscopy, he had a small ascending colon polyp and malignant appearing ulcerated and necrotic tissue at the gastroesophageal junction (Figure 1) concerning esophageal cancer based on the appearance. Biopsies of the mass were obtained, which showed mucosa with chronic inflammation and bacterial colonies of actinomycetes with necrotic material (Figure 2). There was no evidence of malignancy. Unfortunately, the patient died a few days later from an unrelated cause and was unable to undergo treatment for his infection. According to a review of the literature, this would have consisted of IV penicillin for a few weeks followed by oral penicillin for six months to one year.

## Discussion

Actinomyces is an anaerobic, gram-positive bacillus often found in the gastrointestinal and genitourinary flora. They are slow-growing, low virulence organisms that take months to manifest as infections and are commonly mistaken for malignancy or more common infections such as tuberculosis, candida, herpes simple virus (HSV), or cytomegalovirus (CMV) [1]. Actinomyces appear as sulfur granules with club-shaped ends and should be differentiated from Nocardia by the acid-fast bacillus strain. Though sulfur granules are classic for actinomyces, they are only present approximately 50% of the time and if absent, do not definitively rule out actinomyces infection [2]. This organism most commonly causes infections in immunocompromised patients and very few cases have been reported in immunocompetent individuals, such as our patient. Actinomyces most commonly cause infection in the cervicofacial region followed by the thorax, abdomen, or pelvis, and there have been few reports of esophageal infections [2]. These infections typically occur after insult to the esophageal mucosa such as prior surgery or instrumentation, trauma, or infection [3]. They can present as submucosal masses, ulcers, fistulas, abscesses, strictures, or draining sinus tracts [2]. Radiologic findings include esophageal wall thickening on CT or mucosal irregularity such as ulceration, strictures, or multiple sinus tracts on double-contrast esophagram [3]. The gold standard means for diagnosis is culture or histology showing filamentous bacteria and classic sulfur granules however it is important to note that recent antibiotic use, co-infection with other organisms, or improper medium can prevent culture growth [3]. This places emphasis on the importance of histology to often make the diagnosis when cultures are negative. The treatment recommendations are four to six weeks of intravenous penicillin followed by long-term oral penicillin for six months to one year. For penicillin-allergic patients, tetracycline or macrolides can be used [2].

One report according to Pillappa et al. discusses a diabetic, middle-aged female who presented with dysphagia and weight loss [1]. She had no prior esophageal injury and was found to have a firm submucosal mass with superficial ulceration and partial luminal obstruction on esophagogastroduodenoscopy. She was diagnosed with a T3N0 esophageal tumor on endoscopic ultrasound after fine-needle aspiration showed inflammatory debris indicative of neoplasm. She underwent Ivor Lewis esophagogastrectomy with pathology unable to produce evidence of neoplastic cells. After different cultures and staining, the patient was diagnosed with an actinomycetoma. She was treated with high dose penicillin for four weeks and then oral amoxicillin for six months [1].

## Conclusions

In conclusion, actinomyces is an anaerobic bacteria that is slow-growing but can cause occult infections leading to fistulas, abscesses, and strictures. It is important to note that these infections can mimic malignancy leading to unnecessary resection such as in one previously reported patient. Patients should therefore undergo biopsy with culture and histology to prevent unnecessary esophageal resections, which may result in high morbidity in comparison to conservative treatment with antibiotics. The clinician should be aware of these cases and the presentation of actinomyces infection, though rare, to prevent patients from undergoing unnecessary and potentially harmful procedures.

## References

[1] Pillappa R, O'Brien TF, Sullivan JL, Weksler B (2016). Esophageal actinomycoses mimicking malignancy. Ann Thorac Surg.

[2] Zhang AN, Guss D, Mohanty SR (2019). Esophageal stricture caused by actinomyces in a patient with no apparent predisposing factors. Case Rep Gastrointest Med.

[3] Welling RD, Cardona DM, Thompson WM (2009). Esophageal actinomycosis: a case report and review of radiographic findings. J Radiol Case Rep.

